# Aptamer-based approaches for sensitive detection and epitope mapping of SARS-CoV-2 spike protein

**DOI:** 10.1016/j.omtn.2025.102790

**Published:** 2025-12-09

**Authors:** Suttinee Poolsup, Elnaz Yaghoobi, Aliaksandra Radchanka, Nandanee Mulloo, Spencer Uguccioni, John Paul Pezacki, Abdullah Khraibah, Aasha Jawad, Gurcharan K. Uppal, Yuxuan Gu, Benjamin Patrick Lapointe, Nico Hüttmann, Zoran Minic, Polina V. Artyushenko, Irina A. Shchugoreva, Anastasia V. Rogova, Felix N. Tomilin, Dmitry Morozov, Anna S. Kichkailo, Olga S. Kolovskaya, Maxim V. Berezovski

**Affiliations:** 1 Department of Chemistry and Biomolecular Sciences, University of Ottawa, Ottawa, ON K1N 6N5, Canada; 2 John L. Holmes Mass Spectrometry Facility, Faculty of Science, University of Ottawa, Ottawa, ON K1N 6N5, Canada; 3 Laboratory for Biomolecular and Medical Technologies, Krasnoyarsk State Medical University Named after Prof. V.F. Voyno-Yasenetsky, Krasnoyarsk 660022, Russia; 4 Laboratory for Digital Controlled Drugs and Theranostics, Federal Research Center KSC SB RAS, Krasnoyarsk 660036, Russia; 5 Nanoscience Center and Department of Chemistry, University of Jyväskylä, 40014 Jyväskylä, Finland; 6 Laboratory of Physics of Magnetic Phenomena, Kirensky Institute of Physics, Krasnoyarsk 660012, Russia

**Keywords:** MT: oligonucleotides: diagnostics and biosensors, DNA aptamers to SARS-CoV-2 S1 subunit, COVID-19 diagnostics, protein binding epitopes, proximity ligation of aptamers, biolayer interferometry assay

## Abstract

The SARS-CoV-2 spike (S) protein, crucial for viral entry, remains a key target for diagnostics and therapeutics amid evolving variants. Here, we describe the selection and characterization of novel DNA aptamers targeting the S1 subunit, including the Omicron strain, via systematic evolution of ligands by exponential enrichment (SELEX) and biolayer interferometry (BLI). Three aptamers—AptS1-tSP4, AptS1-tSP10, and AptS1-tSP11—exhibited nanomolar dissociation constants (14–59 nM), with AptS1-tSP10 demonstrating good selectivity over MERS-CoV and robust binding in human saliva and pseudovirus samples. Integration with proximity ligation assay and qPCR (PLA-qPCR) achieved a detection limit of 3 pM, surpassing many antibody-based methods. Mass spectrometry-based epitope mapping identified the receptor-binding domain (RBD) peptide VGGNYNYLYR as the primary binding site for AptS1-tSP10. Molecular dynamics and quantum mechanics simulations revealed stable interactions through hydrogen bonding and π-π stacking with neutral residues in both open and closed spike conformations, independent of variant mutations. These multifunctional aptamers offer a versatile platform for ultrasensitive, epitope-specific SARS-CoV-2 detection and pave the way for nucleic acid-based therapeutics to combat viral infections.

## Graphical abstract

**Figure:**
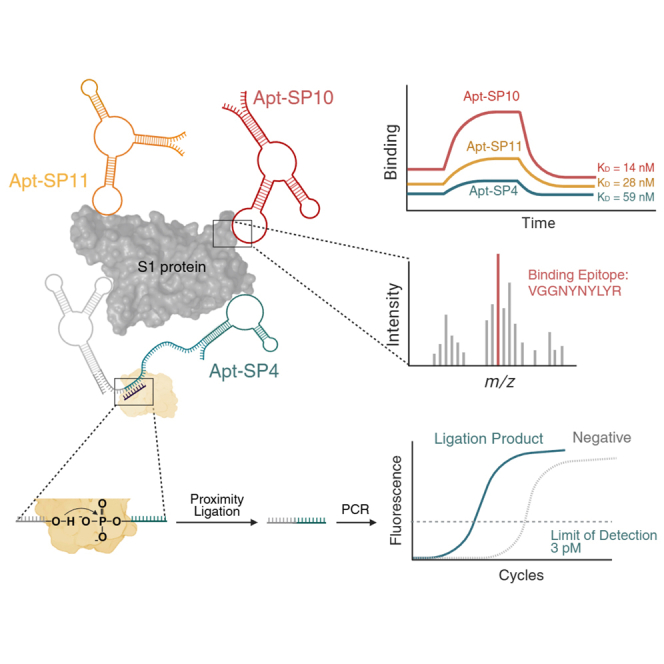

## Teaser

Berezovski and colleagues developed three DNA aptamers with high affinity for the receptor-binding domain of the SARS-CoV-2 S1 protein. Their integration into a proximity ligation assay enabled picomolar detection of the spike protein, demonstrating a highly sensitive and specific nucleic acid-based platform for viral diagnostics.

## Introduction

The use of aptamer technology in molecular diagnostics and therapeutics has increased significantly during the COVID-19 pandemic, particularly in providing several alternative methods to detect the viral proteins from SARS-CoV-2 and to block the viral entry through the interaction of the spike (S) protein and the human receptor. The S protein is the largest transmembrane glycoprotein among the structural proteins of SARS-CoV-2 protruding from the viral surface. The S protein comprises two functional protein subunits, including the S1 and S2 subunits. The S1 subunit is composed of the receptor-binding domain (RBD) showing a high affinity to the human angiotensin-converting enzyme 2 (ACE2) on the host surface receptor, and the S2 subunit mediates the cell fusion and the integration of the viral membrane (E and M proteins) into the host cell membrane rendering the entry of the virus into the cells.^1^ The S, S1, and RBD proteins were widely targeted for COVID-19 diagnostics and therapeutics, including neutralizing antibodies and other viral inhibitors. Although several antigen/antibody-based approaches were studied and developed over the past three years for the diagnosis, some improvements need to be addressed, such as accuracy, sensitivity, easy-to-use, cost-effective and less time-consuming.^2^ Particularly, antibodies have been mainly used as a capture ligand to their target antigens; however, the need for chemical modifications and thermal stability can create obstacles when developing the antigen-detecting platform (e.g., biosensors, nanomaterials, and fluorescence-labeled techniques), these could be obstacles to implement the antibodies on some cutting-edge technologies.^3^ Alternatively, an aptamer, a single-stranded nucleic acid (DNA or RNA) that folds into secondary structures, can function similarly to an antibody in terms of molecular recognition elements. The enrichment of the aptamers with high affinity to their targets is obtained from a randomized DNA/RNA library via the process called systematic evolution of ligands by exponential enrichment (SELEX), meaning that the aptamers can be chemically synthesized and precisely modified while still maintaining their stability and affinity with less batch-to-batch variations, unlike antibodies.^4^^,^^5^

Several RBD- and S1-targeting aptamers have been reported previously, including 1C, 4C, SNAP1.50, SNAP1.66, MSA1T, and MSA5T, and have been implemented on label-free sensing platforms, such as surface plasmon resonance (SPR) and biolayer interferometry (BLI) for real-time detection of recombinant SARS-CoV-2 proteins.^6^^,^^7^^,^^8^^,^^9^^,^^10^^,^^11^ For instance, the 1C aptamer applied on a BLI sensor exhibited a limit of detection (LOD) of ∼250 nM,^7^ which was later improved to 37 nM on an SPR chip.^8^ Other aptamers, including SNAP1.50, SNAP1.66, MSA1T, and MSA5T, were evaluated using BLI and SPR and further integrated into multi-step colorimetric detection platforms, achieving pico- to femtomolar sensitivity.^9^^,^^10^ Despite these advances, many of these aptamers display relatively high K_D_ values, which may limit detection sensitivity in rapid or simplified assays and reduce suitability for early-stage disease diagnostics. Moreover, the multi-step amplification and complex biosensor fabrication required for ultra-low detection introduce additional challenges for routine implementation.^9^^,^^10^ Taken together, these limitations indicate that there remains substantial room for improvement in aptamer affinity and assay design. Specifically, developing new aptamers with lower K_D_ and compatibility with straightforward, rapid detection formats could enhance sensitivity, simplify assay workflows, and broaden applicability for practical SARS-CoV-2 detection.^11^

Regardless of the published sequences of the aptamers against the S1 protein of SARS-CoV-2, only two aptamers, namely ApTOLL targeting toll-like receptor 4 for COVID-19 therapeutics and AptamerX used for saliva-based COVID-19 diagnosis targeting the S protein, have previously been in clinical trials.^12^ In addition to developing aptamer-based COVID-19 diagnostics by improving the sensitivity of existing analytical methods, there are pitfalls in understanding the binding mechanisms of the previously reported aptamers to their targets, especially those against the S1 protein, due to a lack of clarifications. Understanding the binding mechanisms through epitope identification and analyzing the structural conformations of the aptamers when binding to their targets can address questions about how aptamers function at the molecular level and potentially overcome obstacles that hinder transitioning aptamer technology from bench to clinical studies.^13^ To our knowledge, only one recent study by Chen and co-workers^14^ has highlighted the advantages of using bioinformatic tools for predicting aptamer-target binding based on their structures and the application of an artificial intelligence (AI) pipeline. Despite advancements in AI for predicting aptamer-target interactions, *in vitro* studies are essential to validate and support the unambiguous findings obtained through *in silico* methods.^15^

In this study, new sequences of aptamers targeting the S1 subunit of SARS-CoV-2 were identified *in vitro*. The selected aptamers were used to measure S1 protein levels using biolayer interferometry (BLI) and proximity ligation of aptamers with qPCR detection (PLA-qPCR). Aptamers showed strong binding affinity to the Omicron variant (B.1.1.529) with minimal cross-reactivity to MERS S1 protein, and they also demonstrated potential binding to SARS-CoV-2 pseudoviruses. Additionally, the specific epitope on the S1 protein of SARS-CoV-2 that the aptamer binds to was identified, surprisingly located within the RBD region. Molecular dynamics and quantum mechanics simulations of the aptamer and S1 protein confirmed that our selected aptamer interacts specifically with amino acid residues in the RBD of SARS-CoV-2, forming hydrogen bonds and π-π stacking interactions. Identifying these binding epitopes may further aid in developing COVID-19 diagnostics and therapeutics to prevent widespread infection and severe illness in future outbreaks.

## Results

The recombinant S1 subunit of the SARS-CoV-2 spike protein was used as the target for the positive selection of ssDNA aptamers through a SELEX procedure, as detailed in the materials and methods and our previous study.^16^ The affinity of six enriched DNA pools was validated using label-free biolayer interferometry (BLI), which demonstrated markedly higher binding responses compared to the initial N40 DNA library (Figure S1). After next-generation sequencing (NGS) and analysis based on established selection criteria,^16^ fourteen representative sequences (Table S1) were identified and categorized into three families by phylogenetic clustering (Figure S2). These aptamer groups were subsequently examined in BLI assays to identify the most efficient binders and assess their affinity and selectivity toward the S1 protein.

### Binding evaluation and K_D_ determination of aptamers to S1 protein

**Figure 1:**
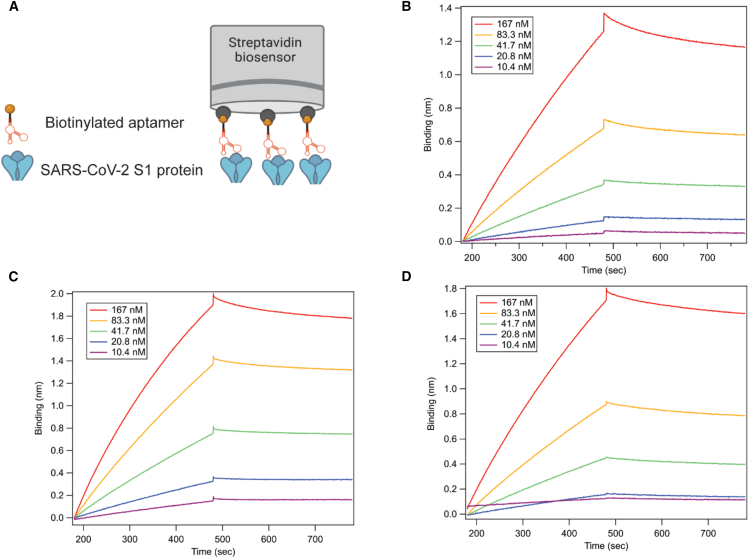
BLI assay of aptamer binding to SARS-CoV-2 S1 protein (A) Schematic overview of the aptamer-based BLI detection setup. The biotinylated aptamer was immobilized on a streptavidin biosensor and incubated with SARS-CoV-2 S1 protein. Binding signals were detected in real time during the association phase (300 s) and dissociation phase (300 s) of the aptamers and the S1 protein. BLI sensorgrams showed a concentration-dependent increase in target binding for the aptamers AptS1-tSP4 (B), AptS1-tSP10 (C), and AptS1-tSP11 (D), after subtracting the signals from the assay buffer with 0 nM of S1 protein as a reference.

The streptavidin biosensor was used to immobilize biotinylated DNA sequences to measure the binding shift to the S1 protein, and the BLI experimental setup is depicted in Figure S3. As a result, aptamer sequences named S1-SP4, S1-SP10, and S1-SP11 showed higher binding shifts than the other eleven aptamer clones under both FW/RW primer hybridization conditions. Consequently, the K_D_s of the truncated sequences of these three aptamers were determined individually by immobilizing the biotinylated aptamers named AptS1-tSP4, AptS1-tSP10, and AptS1-tSP11 on a streptavidin biosensor (Figure 1A). Then, the SARS-CoV-2 S1 protein was spiked into the assay buffer at various concentrations of 10.4, 20.8, 41.8, 83.3, and 167 nM, and control assays without either adding the S1 protein or the aptamer were included to test for non-specific binding. The kinetic binding assay, using the default 1:1 binding model in Octet N1 software, yielded K_D_ values of 59 ± 2.7, 14 ± 2.1, and 28 ± 1.3 nM for AptS1-tSP4 (Figure 1B), AptS1-tSP10 (Figure 1C), and AptS1-tSP11 (Figure 1D), respectively, with an acceptable curve fit (R^2^ = 0.99) (Table 1).

**Table 1:** K_D_s and sequences of truncated aptamers (AptS1-tSP4, AptS1-tSP10, and AptS1-tSP11) binding to SARS-CoV-2 S1 subunit protein

Aptamer	Aptamer sequence	K_D_ (nM)	*k*_a_ (1/M·s)	*k*_d_ (1/s)
AptS1-tSP4	CACGTAATGCCTAACTCTTTTTGTGTTTGCGATCTTTGCACATAGCAT	59 ± 2.7	1.2 × 10^4^	6.8 × 10^−4^
AptS1-tSP10	CACACTTTCTGCCCGCCTTCTCCCTCCGTTCCCCTCCCCG	14 ± 2.1	2.8 × 10^4^	4.0 × 10^−4^
AptS1-tSP11	ATGTCCTCGCACACCCAAACGCACTCATCTCCCCACCCATGCATA	28 ± 1.3	1.1 × 10^5^	5.1 × 10^−4^

The S1 protein was partitioned in the assay buffer while loading onto the aptamer-coated streptavidin biosensor in the association step. The kinetic binding parameters of the aptamers to SARS-CoV-2 S1 protein were calculated using a 1:1 binding model. The sequences of these selected aptamer candidates were shortened based on the prediction of their secondary structures obtained from RNAstructure web server.^17^

### Proximity ligation of aptamers and qPCR detection of S1 protein

**Figure 2:**
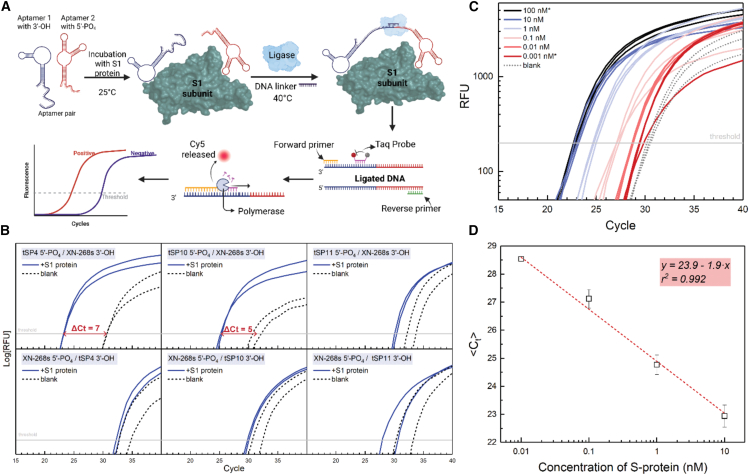
Proximity ligation of aptamers with qPCR detection (PLA-qPCR) of S1 protein (A) Schematic diagram of the PLA-qPCR workflow, showing aptamer-target binding, ligation, and PCR amplification of the ligated product. (B) Screening of aptamer pairs for PLA-qPCR efficiency. Upper panels show PCR amplification of ligated aptamer pairs AptS1-tSP4, AptS1-tSP10, AptS1-tSP11 with 5′-phosphate, and XN-268s with 3′-hydroxyl. Lower panels display PCR amplification with reversed functionality: AptS1-tSP4, AptS1-tSP10, and AptS1-tSP11 with 3′-hydroxyl, and XN-268s with 5′-phosphate. Each sample is tested in duplicate. Significant cycle differences between positive and negative samples are seen for AptS1-tSP4 (5′-PO_4_) with XN-268s (3′-OH) and AptS1-tSP10 (5′-PO_4_) with XN-268s (3′-OH). (C) PLA-qPCR curves for detection of the S1 protein (1 pM–100 nM) using the AptS1-tSP4 (5′-PO_4_) and XN-268s (3′-OH) pair. Concentrations of 100 nM and 0.001 nM (marked with asterisks) are outside the linear range and were excluded from the calibration curve. Each sample is analyzed in triplicate. (D) Calibration curve for S1 protein (10 pM–10 nM) based on average cycle threshold (Ct) values from C.

BLI experiments confirmed the strong binding affinity of three selected aptamers, providing a foundation for developing a PLA-qPCR assay to detect S1 protein with high sensitivity. Briefly, in PLA-qPCR, two aptamers bind closely to a protein, allowing a linker to connect their 3′-hydroxyl and 5′-phosphate ends. A T4-ligase then joins these ends, producing a PCR-detectable product (Figure 2A). Initial attempts to perform PLA-qPCR using only the aptamers selected in this work did not produce a measurable difference in Ct values between positive and negative samples; these data are provided in the Supporting Information (Figure S5). To develop the method, six aptamer pairs were tested (Table S4), including the three aptamers selected in this work (AptS1-tSP4, AptS1-SP10, and AptS1-SP11) and one published aptamer, XN-268s, which was chosen because it was selected under the same SELEX conditions as our aptamers (DPBS buffer with Ca^2+^/Mg^2+^) and has a reported low K_D_ 4.26 nM, indicating high binding affinity.^18^ At a 10 nM S1 protein concentration in the ligation mixture, two pairs—AptS1-tSP4 with 5′-PO_4_/XN-268s with 3′-OH (ΔC_t_ = 7) and AptS1-tSP10 with 5′-PO_4_/XN-268s with 3′-OH (ΔC_t_ = 5)—exhibited significant PCR cycle differences, demonstrating superior performance over other pairs (Figure 2B).

The AptS1-tSP4 with 5′-PO_4_/XN-268s with 3′-OH pair showed a larger cycle difference between positive sample and blank and was chosen for a titration experiment to determine LOD for S1 protein. This curve revealed a linear detection range for protein concentrations from 10 pM to 10 nM (Figures 2C and 2D). The limit of detection (LOD) was determined to be 3 pM, equivalent to 4.8 × 10^5^ particles in 20 μL of sample, based on cryo-EM,^19^ cryo-ET,^17^^,^^19^ and EM morphometry^17^^,^^20^ estimates of ∼25 spike-protein trimers per virion.

### Selectivity of aptamer binding to S1 proteins

**Figure 3:**
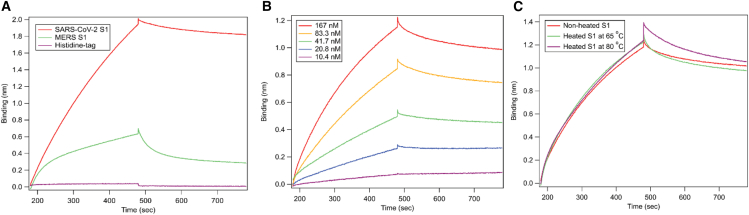
Selectivity of AptS1-tSP10 aptamer binding to SARS-CoV-2 S1 protein and detection of the SARS-CoV-2 S1 protein of the Omicron variant (A) BLI sensorgrams showing the association and dissociation of the AptS1-tSP10 aptamer with 20 μg/mL of wild-type SARS-CoV-2 S1 and 20 μg/mL of MERS-CoV S1 in PBST buffer, as well as 20 μg/mL of hexahistidine peptide as a control for non-specific binding. (B) BLI sensorgrams demonstrating the increased signals of the AptS1-tSP10 aptamer binding to the concentration-dependent S1 protein of the Omicron variant after subtracting the assay buffer with 0 nM S1 protein as a reference. (C) The binding affinity of the AptS1-tSP10 aptamer to the S1 proteins of the Omicron variant was observed under heat treatment at 65°C for 15 min and 80°C for 5 min.

Considering the strong affinity of the AptS1-tSP10 aptamer for the S1 protein, which showed the lowest K_D_ among the selected sequences, this aptamer was used in BLI studies. The selectivity of AptS1-tSP10 binding to the SARS-CoV-2 S1 protein was evaluated by comparing the BLI binding shift (nm) of the aptamer to the S1 subunit of MERS-CoV and a hexahistidine peptide tagged at the C-terminal of the S1 protein (Figure 3A). No binding of the AptS1-tSP10 aptamer to the hexahistidine peptide was observed. Notably, this aptamer displayed stronger binding to the SARS-CoV-2 S1 protein than to the MERS-CoV S1 protein, with about 30% binding to the AptS1-tSP10 aptamer (Figure 3A). Additionally, an affinity study of AptS1-tSP10 for the Omicron variant was conducted using the Octet N1, revealing a K_D_ of 16 ± 0.7 nM (Figure 3B). The effect of heat treatment on the binding affinity of AptS1-tSP10 to the Omicron S1 protein was also examined after heating the protein at 65°C for 15 min and at 80°C for 5 min. As shown in the BLI sensorgrams in Figure 3C, heating the S1 protein at these temperatures did not reduce the binding signal of AptS1-tSP10 to the heat-inactivated Omicron S1 protein. This suggests a high stability of the Omicron S1 protein, as detected by the AptS1-tSP10 aptamer using the BLI approach.

### Detection of SARS-CoV-2 S1 protein of the Omicron variant (B.1.1.529) in human saliva and SARS-CoV-2 pseudoviruses using AptS1-tSP10 aptamer-based BLI

**Figure 4:**
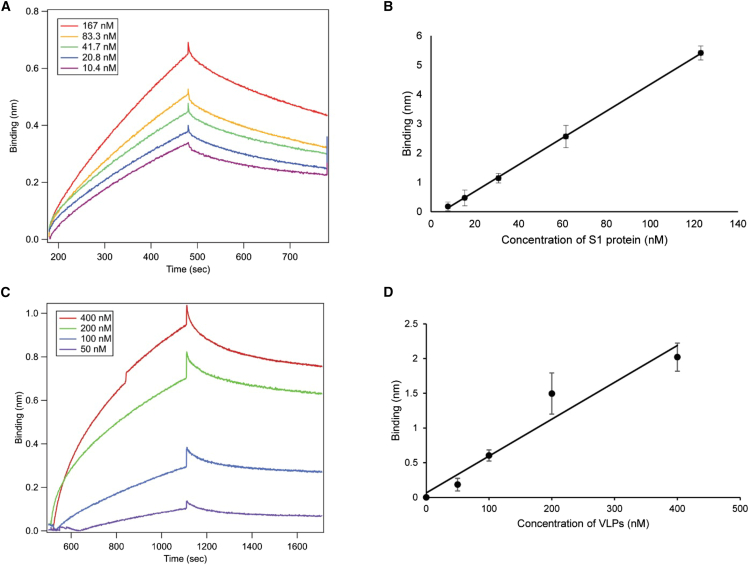
Aptamer-based BLI detection of SARS-CoV-2 S1 protein in human saliva (A) and (C) BLI sensorgrams showing the binding assay of AptS1-tSP10 aptamer with the S1 protein spiked in human saliva and with pseudoviruses of SARS-CoV-2, respectively, using 2-fold dilutions to determine the K_D_. (B) and (D) Linear regression curves of the binding shifts corresponding to the S1 protein in the 10.4–167 nM range and the pseudoviruses in the 50–200 nM range, respectively.

The AptS1-tSP10 aptamer-based BLI assay was used to detect the SАRS-CoV-2 S1 protein of the Omicron variant in human saliva. The biotinylated aptamer was loaded onto a streptavidin biosensor, and various concentrations of the Omicron S1 protein were prepared in 10% saliva diluted in PBST assay buffer to assess binding to the AptS1-tSP10 aptamer. The binding measurement took place within 14 min per sample, and a control assay was performed with 10% saliva without the target S1 protein. As a result, the AptS1-tSP10 aptamer could detect the S1 protein within a concentration range of 10.4–167 nM spiked into pooled human saliva, and the K_D_ was calculated at 127 ± 2.5 nM (Figure 4A). Additionally, the non-specific binding was tested as a control. The highest concentration of S1 protein in the saliva assay buffer did not increase binding to the bare streptavidin biosensor without the AptS1-tSP10 aptamer, indicating that non-specific binding was negligible for this aptamer-based detection of the S1 protein.

The diagnostic evaluation of the AptS1-tSP10 aptamer-based BLI to detect SARS-CoV-2 pseudoviruses was also performed in the assay buffer. The binding shifts on the aptamer-based BLI during the interaction with the pseudoviruses indicated a dose-dependent response, and the K_D_ was determined to be 112 ± 4.5 nM (Figure 4C). The LOD of this aptamer-based BLI approach was calculated as 19 nM for detecting the S1 protein, and 33 nM or 5 × 10^9^ particles/mL of the pseudoviruses (Figures 4B and 4D).

### Identification of the aptamer binding epitope of S1 protein by nLC-MS/MS

**Figure 5:**
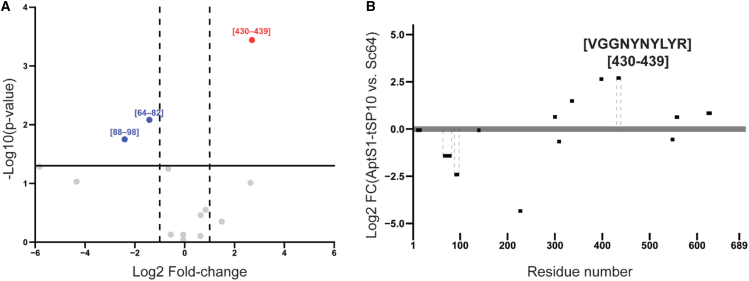
Identification of an epitope peptide binding to AptS1-tSP10 aptamer by nLC-MS/MS (A) Volcano plot displaying fold-change (*x* axis) and statistical significance (*y* axis) of individual peptides based on label-free quantification (LFQ) intensity. Red points highlight peptides of interest that appear in the eluted peptides from the AptS1-tSP10 aptamer with insignificantly higher abundance than those from the control DNA. (B) Peptide plot showing log2 fold-change differences along the SARS-CoV-2 S1 sequence (denoting position in the SARS-CoV-2 Spike protein, UniProt: P0DTC2). The peptide that was significantly more abundant in the fractions eluted from the aptamers compared to those from the control DNA was identified by a paired *t* test and marked with dotted lines.

To determine the peptide sequences of binding epitopes on the S1 protein, we performed the experiment using the same protocol reported in our previous study.^16^ Briefly, the AptS1-tSP10 tagged with biotin was incubated with the S1 subunit protein for 30 min at 25°C, and the unbound biotinylated aptamer was removed using a 30 kDa centrifugal filter. The aptamer-bound protein complex was trypsinized at 37°C for 30 min and captured on streptavidin magnetic beads before eluting the aptamer-bound peptides. The intensities of eluted peptides from the AptS1-tSP10 aptamer were statistically compared to those from the control (Sc64), non-specific scrambled 64 nt DNA sequence, with a *p* value below 0.05. The differences in individual peptide abundance were shown as volcano plots and visualized along the protein sequences. Figure 5 shows the peptide (VGGNYNYLYR), named SA10, with the highest abundance at positions 430–439, as a binding epitope of the AptS1-tSP10 aptamer, compared to the negative control. Therefore, this peptide position could be a potential binding epitope of the AptS1-tSP10 aptamer on the S1 subunit of the SARS-CoV-2 spike protein.

### Molecular modeling of the aptamer-protein binding

**Figure 6:**
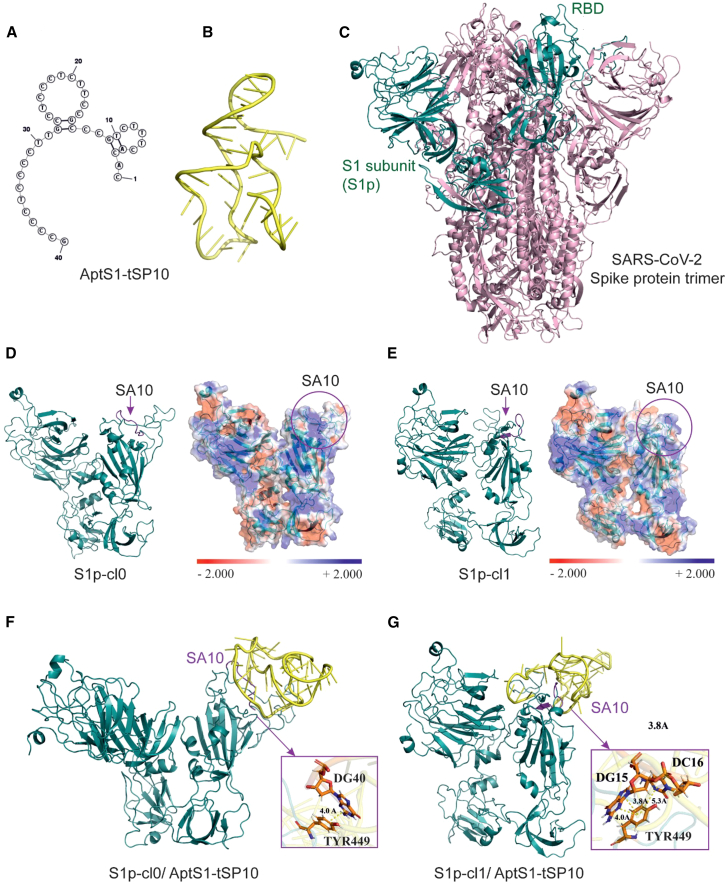
Structures of the AptS1-tSP10 aptamer (yellow), the target S1 protein (green), and S1p/AptS1-tSP10 complexes (A) and (B) show the secondary and tertiary structures of the AptS1-tSP10 aptamer, respectively; (C) is a 3D model of the SARS-CoV-2 spike protein (pink), with the cyan color highlighting the target S1p subunit on the spike protein for *in vitro* and *in silico* investigations. This subunit includes the RBD; (D) and (E) show tertiary structures of the target protein obtained after clustering MD trajectories, with the charge distribution map included alongside the SA10 binding sites indicated in purple within the pink circle; (F) and (G) display tertiary structures of the aptamer/S1p complex, with the pink squares illustrating examples of strong interactions between TYR449 of the SA10 binding site and the nucleotides of the aptamer, where the main interaction component is the dispersion energy (E disp.). Distances between residues are given in angstroms, Å.

Based on the nucleotide sequence of the AptS1-tSP10 aptamer, its secondary structure was modeled using the mFold server (Figure 6A). The corresponding tertiary structure was then designed with the SimRNA and VMD programs. The resulting spatial structure was subjected to 200 ns molecular dynamics simulations, followed by clustering analysis of the MD trajectories. MD simulations were carried out using the GROMACS-2021 package. The specific parameters of the calculations are detailed in the Methods section. The representative cluster was selected as the aptamer’s spatial structure for further studies (Figure 6B).

To obtain the 3D model of the target protein, we selected the structure of the SARS-CoV-2 spike protein (6ZP0) and isolated the part of the protein corresponding to the S1 subunit used in the *in vitro* experiments (Figure 5C, green). This portion of the S1 subunit includes the receptor-binding domain (RBD). Two clusters of the target protein showing the most stable conformations from MD simulations and clustering analysis were identified, representing the open and closed conformations of the protein in solution. Therefore, we focused only on these two structures and named them S1protein_cluster 0 (S1p_cl0) and S1protein_cluster 1 (S1p_cl1) (Figures 6D and 6E, respectively) for further analysis.

According to the experimental results, a protein sequence VAL445, GLY446, GLY447, ASN448, TYR449, ASN450, TYR451, LEU452, TYR453, and ARG454 called SA10 was identified as a binding site of the AptS1-tSp10 aptamer on the S1 subunit protein. Notably, this SA10 binding region, located on the surface of the S1 subunit, can be accessed by the aptamer in both clusters (Figures 6D and 6E, purple color). Charge distribution maps for the protein clusters were modeled using the APBS plugin and shown in Figures 6D and 6E. Molecular docking was also performed with the HADDOCK web server for both protein clusters S1p_cl0 and S1p_cl1. The SA10 sequence was considered as a binding site in the docking simulations, and the models of the highest-scoring protein-aptamer complexes were selected for further MD simulations. The SA10 sequence was considered as a binding site in the docking simulations, and the models of the highest-scoring protein-aptamer complexes were selected for further MD simulations. Structures of the aptamer complexes with S1p_cl0 and S1p_cl1 were obtained through clustering MD trajectories (Figures 5F and 5G, respectively). Hydrogen bonds between the aptamer nucleotides and amino acids in the SA10 binding region (Table S2) were analyzed using the VMD program, involving GLY446, ASN448, and ASN450 of S1p_cl0, and G447 of S1p_cl1 conformations.

To further explore how the amino acids at the SA10 binding sites interact with the nucleotides of the AptS1-tSP10 aptamer, quantum chemical calculations were performed using the GAMESS(US) program. These calculations aimed to identify which amino acids and nucleotides mainly contribute to the interaction between the aptamer and the binding region on the S1 protein. Pair interaction energy decomposition analysis (PIEDA), combined with the two-body fragmented molecular orbital (FMO) method, was used to estimate pair interaction energies and analyze these interactions in terms of electrostatic, exchange-repulsion, charge-transfer, dispersion, and polarization energies for large molecular systems. Dispersion interactions (also known as London dispersion) are considered the primary contributors to attractive forces and play a key role in molecular aggregation and recognition.^21^ Residue interactions with pair interaction energies (PIE) ≤ −3 kcal/mol are regarded as significant mediators of the interactions between the amino acids on the SA binding region and the nucleotides on the aptamer. As shown in Table S3, the lowest calculated interaction energies for amino acids, including asparagine, glycine, and valine of the S1p-cl0 cluster, as well as tyrosine of the S1p_cl1 cluster on the SA10 binding site of the S1 protein, indicate that these amino acids are actively involved in binding to the AptS1-tSP10 aptamer. The total interaction energy was −277.1 kcal for the open conformation and −254.73 kcal for the closed conformation of the S1 protein. It is evident that strong interactions occur through both electrostatic (pink) and dispersion (blue) forces within the binding site. In both protein conformations, tyrosine residues primarily contribute to dispersion through π-π stacking interactions (Figures 6F and 6G).

## Discussion

The development of aptamer-based antigen detection has significantly impacted the rapid spread of SARS-CoV-2 during the pandemic. Several aptamers targeting the spike protein have been previously reported for COVID-19 diagnosis and therapeutic purposes, such as blocking the virus’s entry by preventing the interaction between the RBD region of the spike protein and angiotensin-converting enzyme-2 (ACE-2), a primary receptor on human epithelial cells.^22^ To diversify the selection of aptamer sequences against the S1 protein, especially those binding to the RBD region, our work identified novel DNA aptamers specifically binding to the S1 protein of SARS-CoV-2. Through stringent affinity screening, the primer binding sites of the 80-nucleotide aptamers were hybridized with 5′-FW and 3′-RW primers tagged with biotin, allowing them to be attached to streptavidin biosensors. This confirmed that the primer binding regions do not play an important role in the aptamer’s binding to the target protein, as discussed in the previous study of aptamers targeting the nucleocapsid protein.^16^

The findings show that the selected aptamers can be effectively used not only in BLI studies but also in the PLA method. While BLI was used to characterize the binding strength and kinetics of the aptamer-protein interaction, PLA-qPCR was subsequently applied to achieve highly sensitive and specific detection, with false-positive signals reduced through dual-probe recognition and true binding events amplified by qPCR.^23^^,^^24^ Specifically, the aptamer pair AptS1-tSP4 with 5′-PO4 and XN-268s with 3′-OH demonstrated a LOD for the S1 protein in the picomolar range, indicating a sensitivity at least two orders of magnitude higher than that of ELISA.^25^ The PLA was performed using a commercially sourced S1 protein diluted in buffer; however, it has not yet been tested in more complex biological samples, such as serum or saliva.

The strong binding aptamer (AptS1-tSP10) with a K_D_ of 14 nM demonstrated high selectivity for SARS-CoV-2 over MERS. Due to the highly positive charges of the S1 protein at a physiological pH (∼ pH 7.4), the weak interaction signal of the AptS1-tSP10 with the S1 subunit of MERS—around 25%–30% binding compared to its binding to SARS-CoV-2—may be caused by electrostatic interactions between the positive charges on amino acid residues and the negative charges of the phosphate groups on the DNA aptamer.^26^ The affinity evaluation of the AptS1-tSP10 aptamer for the Omicron variant and viral particle-like structures also highlighted its versatile recognition of diverse SARS-CoV-2 variants and intact structures, yielding K_D_ values in the nanomolar range. This sparked our curiosity about how the aptamer might interact with the S1 subunit of SARS-CoV-2. According to results from the affinity-MS study, the AptS1-tSP10 aptamer was used as a capture molecule on the S1 protein before trypsin digestion, and the peptide profile was generated using nLC-MS/MS. Notably, a potential binding epitope with 10 amino acids (SA10) was identified and located within the receptor-binding domain (RBD) of the S1 protein.

To explore the molecular interactions between the DNA aptamer and the amino acid sequence of the binding epitope, the tertiary structures of the aptamer and the S1 protein were modeled, and their interaction interface was analyzed using molecular dynamics and quantum mechanics methods. The 3D simulation notably showed a strong binding of the AptS1-tSP10 aptamer to amino acid residues, such as Asn, Gly, Val, and Tyr in the RBD region of the S1 protein, in both open and closed conformations. Due to the neutral net charge of these residues at physiological pH, a significant dispersion contribution was observed between the aptamer and aromatic amino acid residues through π-π stacking.^27^ At the aptamer-protein interface, these interactions occurred mainly in the SA10 binding region, which largely contributed to the binding affinity and specificity of the aptamer. The binding affinity of the aptamer to the purified S1 subunit protein in human fluids like saliva (K_D_ ∼127nM) was slightly different from that to the S1 on the intact pseudovirus envelope protein (K_D_ ∼112 nM). This difference supports the idea that the AptS1-tSP10 aptamer maintains strong binding to the S1 protein even after heating, which imitates the heat inactivation process during clinical sample handling.

The strong feature of the aptamer-based BLI, which can be used with crude samples, was employed to validate the detection of the S1 protein in human saliva. This was accomplished using the AptS1-tSP10 aptamer immobilized on the biosensor, which still yielded K_D_ and LOD values in the nanomolar range even in complex bioanalytical samples like human saliva. Comparing the detection sensitivity across different methods reported in previous studies^8^^,^^18^^,^^28^^,^^29^ is quite challenging when considering K_D_ and LOD values. The detection limit of the aptamer-based BLI was not shown to be the most sensitive analytical approach, but this method remains a promising alternative because of its simplicity and robustness in detecting the SARS-CoV-2 spike protein in clinical samples. Therefore, we used PLA with PCR detection to improve LOD (3 pM), which is equivalent to 4.8·10^5^ particles in 20 μL of sample.

In conclusion, three aptamers—AptS1-tSP4, AptS1-tSP10, and AptS1-tSP11—were chosen to target the S1 subunit and successfully used in BLI and PLA analytical methods, showing their versatility as universal detection agents. AptS1-tSP10 specifically binds to the S1 subunit of the SARS-CoV-2 spike protein and the Omicron variant (B.1.1.529) with low nanomolar K_D_. Notably, molecular dynamics and quantum mechanics simulations of the aptamer binding to the identified peptide-binding epitope (SA10) confirmed strong interactions between the aptamer and neutral-charged amino acids through hydrogen bonding and dispersive forces, in both open and closed conformations of the spike protein. These findings underscore the potential of this aptamer for developing more sensitive analytical techniques, as well as its use in immunoprecipitation and therapeutics for viral infectious diseases.

## Materials and methods

The DNA (N40) Library, 5′-CTCCTCTGACTGTAACCACG-(N40)-GCATAGGTAGTCCAGAAGCC -3′, forward primer (5′-CTCCTCTGACTGTAACCACG-3′), reverse primer (5′-GGCTTCTGGACTACCTATGC-3′), modified aptamers, linker, probe and sets of corresponding primers for PLA assay (Table S4) were purchased from Integrated DNA Technologies (Newark, NJ, USA). The his-tagged SARS-CoV-2 S1 proteins of wild-type (Cat. no. S1N-C52H3) and Omicron/B.1.1.529 (Cat. no. S1N-C52Ha) were purchased from ACRObiosystems. Salt-tolerant T4-DNA ligase (Cat. no. M0467S) and Taq 2× Master Mix (Cat. no. M0270L) were purchased from New England Biolabs. Phosphate buffer saline (PBS) without Ca^2+^-Mg^2+^ (Cat. no. 10010031) and DPBS with Ca^2+^-Mg^2+^ (Cat. no. 14040141) were purchased from Gibco. tRNA (Cat. no. 15401-011) was purchased from Thermo Fisher Scientific. BSA (Cat. no. A9418), Tween 20 (Cat. no. 9005-64-5), Span80 (Cat. no. 85548), Tween80 (Cat. no. P4780), Triton X-100 (Cat. no. T9284) and mineral oil (Cat. no. M8410) were purchased from Sigma-Aldrich. Microcon-10kDA Centrifugal filter (Cat. no. MRCPRT010) was purchased from Millipore. Ni-NTA HisSorb Strips (Cat. no. 1002478) were purchased from Qiagen. Low binding tubes were purchased from Eppendorf (Cat. no. 022431064) and low binding tips were purchased from Corning (Cat. no. 4154). All buffers should be filtered through a 0.22 μm filter before use. The stock solution of S1 protein was prepared according to the SDS protocol from ACROBiosystems by diluting the commercial lyophilized powder in LC-MS grade water and storing it at −70°C for no longer than 3 months.

### Selection of DNA aptamers targeting SARS-CoV-2 S1 protein

The aptamer selection method was slightly altered from the previous approach used for selecting aptamers targeting the nucleocapsid of SARS- CoV- 2.^15^ Two nanomoles of ssDNA (*N* 40) library were mixed in 300 μL of DNA-heating buffer (DPBS with Ca ^2+^ and Mg ^2+^), then heated at 95° C for 5 min and cooled on ice for 10 min. Next, the heat-folded DNA library was prepared in the selection buffer (PBS with Ca^2+^ and Mg^2+^, 2 μg/μL BSA, 0.2 μg/μL tRNA). For the first round of selection, the heat-folded library underwent negative selection by adding it to each well of the 8-well Ni-NTA strip and incubating at room temperature on an orbital shaker for 30 min. During six rounds of aptamer selection, negative selections were repeated twice, including in the first round (as described previously) and the fourth round. Before starting the fourth round, negative selection was performed by incubating the enriched DNA pool from the third round with non-targeted protein-coated 8- well Ni- NTA strips on the orbital shaker at room temperature for 30 min. The non-target unbound DNA pool was then transferred to strips coated with the S1 protein and incubated on the orbital shaker at room temperature for 1 h. After DNA-protein incubation, 200 μL of washing buffer (DPBS with Ca^2+^ and Mg^2+^ plus 0. 05% Tween 20) was added to each well to remove unbound DNA on the protein-coated strips, and the supernatant was discarded. The washing step was repeated twice. The stringency of subsequent rounds could be increased by adding more washings or reducing the DNA-protein incubation time. Next, 200 μL of DPBS with Ca^2+^ and Mg^2+^ was added to each well to prevent Tween 20 carryover into the PCR reaction. Following this, 200 μL of nuclease-free water was added to each well and incubated at 95°C for 10 min to elute the S 1 protein-bound DNA from the strip, after which the eluted DNA was transferred to a 1.5 mL centrifuge tube and cooled at room temperature. To concentrate the DNA, the eluted pool was transferred to a 10 kDa centrifugal filter and centrifuged at 14,000 rpm for 20 min until the final volume reached 100 μL on the filter.

The concentrated DNA pool obtained from the selection steps was subjected to asymmetric emulsion PCR with the FW: RW primer ratio of 20:1. At the same time, the emulsion oil mixture was prepared by homogenously mixing 4.5% span80, 0.4% Tween80, and 0.05% Triton X-100 in mineral oil. Then, the 200 μL of asymmetric PCR mixture was slowly added dropwise to 400 μL of emulsion oil while stirring. After 5-min stirring, 50 μL of emulsion PCR mixture was aliquoted into each PCR tube. After optimization of the number of PCR cycles, the PCR reaction was amplified for 25 cycles by using the following PCR program: 98°C for 30 s, 25 cycles at 98°C for 30 s, 56°C for 15 s, 72°C for 15 s, and held at 4°C. Then, the asymmetric ePCR product obtained from the previous steps was extracted and purified by using isopropanol and a PCR cleanup kit.

After six rounds of selection, the sequences of the ssDNA from the enriched pools were obtained from the next-generation sequencing, and the raw data were analyzed by following the guidelines from FASTAptamer.^30^ The similarity and differentiation of individual DNA sequences were categorized using the multiple sequence alignment tool on Clustal Omega.^31^ The secondary structures of the selected aptamers were then predicted using the RNAstructure web server.^32^

### Affinity screening of DNA aptamers by bio-layer interferometry

For the binding test of the selected aptamers against the SARS-CoV-2 spike S1 subunit, streptavidin biosensors were used on the Octet N1 (Figure 3). To prepare biotinylated aptamers, the 80 nt-long DNA sequences were hybridized with biotin-labeled FW and RW primers separately in DPBS at 95°C for 5 min, then the temperature was gradually lowered to 25°C over 30 min and held at 4°C for 30 min on the thermocycler. The ratio of biotin-labeled primers to unlabeled 80 nt DNA sequences was 1:1 (400 nM:400 nM) in 100 μL of the mixture. Next, the biotin-FW-hybridized and biotin-RW-hybridized DNA sequences were prepared in the optimized assay buffer (PBST, DPBS with 0.5 mM MgCl_2_ and 1 mM CaCl_2_, pH 7.4, supplemented with 0.2% BSA and 0.05% Tween 20) for the affinity test performed on the Octet N1. Initially, 400 nM of the biotinylated aptamer was heat-folded at 95°C for 5 min, then cooled on ice before loading as a ligand onto a streptavidin biosensor. The aptamer-loaded streptavidin biosensor was then dipped into 20 μg/mL of the S1 protein prepared in the assay buffer, with 300 s for association and 300 s for dissociation to evaluate the affinity of the selected aptamers to the S1 protein. The initial N40 DNA library and the scrambled DNA were also tested as negative controls.

The binding assay was conducted by immobilizing the aptamers on the biosensor tips to detect the presence of the S1 protein in the assay buffer. Serial dilutions of the S1 protein (10.4, 20.8, 41.7, 83.3, and 167 nM) were spiked into the optimized assay buffer (PBST, DPBS with 0.5 mM MgCl_2_ and 1 mM CaCl_2_, pH 7.4, with 0.2% BSA and 0.05% Tween 20). Detection was carried out by dipping a streptavidin biosensor (Sartorius, Bohemia, NY), loaded with the biotinylated aptamer, into the buffer containing the spiked S1 protein. The assay was performed at 25°C, with 300 s for association and 300 s for dissociation. The buffer was used to wash away unbound biomolecules from the biosensor tip during the baseline step for 60 s. The apparent dissociation constant (K_D_) for each aptamer was calculated using Octet N1 software (version 1.3.0.5) with a 1:1 global binding model. In addition to testing binding to SARS-CoV-2 wild-type S1 protein, the affinity of AptS1-tSP10 to the Omicron variant S1 protein (10.4, 20.8, 41.7, 83.3, and 167 nM) was also evaluated under the same conditions. Furthermore, the K_D_ of AptS1-tSP10 binding to pseudoviruses (50, 100, 200, and 400 nM) was measured using Octet N1 with 600 s for association and 600 s for dissociation. The limits of detection (LODs) of the AptS1-tSP10 based BLI assay for the S1 soluble protein and the intact protein on pseudoviruses were determined from the standard deviation (SD) of the binding curves and the slope of binding (nm) versus target concentration (nM), calculated as LOD = 3.3SD/slope.

### Cross-reactivity assessment of aptamers to SARS-CoV-2 S1 protein

The cross-reactivity of AptS1-tSP10 was also investigated by testing the selectivity of the aptamer to the S1 protein of SARS-CoV-2 compared to MERS-CoV, a non-target protein, at a concentration of 167 nM. The AptS1-tSP10 aptamer was prepared in the assay buffer as described previously. The binding assay was performed at 25°C with a 60-s baseline, 300 s for the association step and 300 s for the dissociation step. The apparent K_D_ values were calculated by Octet N1 software (version 1.3.0.5) using a 1:1 global binding model. To verify the non-specific binding to the protein tag, the hexahistidine peptide was also included as a control in the binding assay.

### Production of pseudovirus

SARS-CoV-2 S pseudovirus was produced following previously published methods.^22^ In brief, HEK293T cells were seeded at 3 million cells per 10 cm dish. The next day, cells were co-transfected with 2 μg of a pcDNA3.1 plasmid containing codon-optimized cDNA for the SARS-CoV-2 S glycoprotein (or an empty pcDNA3.1) and 4 μg of HIV-NL4.3 ΔEnv Vpr Luciferase Reporter Vector (pNL-4.3.Luc.R-E). Transfections used a ratio of 1.2 μL lipofectamine 2000 per μg of plasmid, with plasmids and lipofectamine 2000 diluted in 12× Opti-MEM, mixed, incubated for 15 min, and then added to the plates. After 72 h, supernatants containing the pseudovirus were collected, centrifuged at 800 g for 5 min to remove cell debris, and passed through a 0.45 μm filter.

To confirm that pseudoviruses were assembled into infectious virions, Huh7 cells were seeded in 24-well plates at 30,000 cells per well. The following day, cells were transduced with 100 μL of supernatant containing pseudovirions. Plates were then centrifuged at 800 g for 1 h for “spinfection,” which promotes efficient viral attachment. After a 48-h incubation, cells were lysed using a 1× Passive Lysis Buffer (Promega). Luciferase activity was measured with a SpexMax M2 plate reader (Molecular Devices). The luciferase signal was normalized to total protein concentration, determined by Bradford assay. Each biological replicate was measured in technical triplicate. Pseudoviruses were considered properly assembled if the signal was at least 50 times higher in Huh7 cells transduced with supernatant from HEK293T cells transfected with the SARS-CoV-2 S glycoprotein plasmid compared to cells transfected with empty pcDNA3.1.

Before detection by western blotting and BLI, supernatants confirmed to contain properly assembled pseudoviruses were pelleted through ultracentrifugation at 27,800 rpm for 3 h in an AH-629 swinging bucket rotor at 4°C. Supernatants with properly assembled pseudoviruses were layered on top of a 30% sucrose cushion (30% sucrose v/w, 100 mM NaCl, 8 mM MgSO_4_, 50 mM Tris-HCl, and pH 7.5) in 17.0 mL thin-walled polyallomer tubes (ThermoFisher). After centrifugation, the pelleted pseudoviruses were collected and used for downstream analyses.

### Cell lysis and western blotting

HEK293T cells that were co-transfected with 2 μg of pcDNA3.1 plasmid containing codon-optimized cDNA for the SARS-CoV-2 S glycoprotein (or empty pcDNA3.1) and 4 μg of HIV-1-NL4-3 ΔEnv Vpr luciferase reporter vector (pNL4-3.Luc.R-E) were lysed 72 h post-transfection in RIPA lysis buffer (25 mM Tris-HCl pH 7.4, 150 mM NaCl, 1% NP-40, 0.5% sodium deoxycholate, and 0.1% SDS). Lysate concentration was determined by DC assay according to the manufacturer’s protocol (BioRad), and 40 μg of cell lysate was loaded and run on 10% TGX Stain-Free FastCast acrylamide gels (BioRad). For purified infectious pseudovirus, 40 μL of pelleted pseudovirus resuspended in RIPA buffer was loaded. Proteins were transferred to a PVDF membrane using the TransBlot Turbo semi-dry transfer system (BioRad), and membranes were blocked for 1 h at room temperature in 5% skim milk in Tris-buffered saline with 0.1% Tween 20 (TBST, pH 7.4). Membranes were then probed with primary antibodies overnight at 4°C, followed by incubation with the appropriate secondary antibody for 1 h at room temperature. Blots were visualized on a ChemiDoc MP (BioRad) using Clarity ECL solution reagent. The results of quality control of the pseudoviruses production are shown in Figure S4.

### Proximity ligation of aptamers with qPCR detection (PLA-qPCR)

A stock solution of SARS-CoV-2 S1 protein (600 μg/mL, 7.80 μM) was diluted to 100 nM in DPBS containing Ca^2+^ and Mg^2+^. Dilutions were made in low-bind tubes using low-bind pipette tips, combining 2.56 μL of 7.80 μM S1 stock, 19.74 μL of 10× DPBS, and 77.7 μL of water (LC-MS grade). Aptamer pairs were folded before incubation with the protein. A 13 μL folding mixture was prepared with 2 μL of 1 nM aptamer pair, 1.8 μL of 10× DPBS, and 9.2 μL of water. The mixture was heated to 95°C for 20 s and cooled to 15°C at 1°C per second using a SimpliAmp Thermal Cycler (Thermo Fisher Scientific). To the folded aptamer mixture, 2 μL of 100 nM S1 protein was added, and the mixture was incubated at room temperature for 30 min. For negative controls, 2 μL of water was added instead of S1 protein. The sequence of all aptamers, a linker, and probes used in PLA-qPCR is shown in Table S4.

A 5 μL ligation mixture was prepared with 1 μL of salt-tolerant T4 DNA ligase, 2 μL of 10× ligation buffer, and 2 μL of 3 nM linker DNA. This was added to the 15 μL aptamer-protein mixture at 4°C in the thermocycler, resulting in a 20 μL reaction volume. Ligation was performed at 40°C for 7 min, followed by ligase inactivation at 95°C for 15 min. The ligated product was cooled to room temperature and diluted 5-fold with 100 μL of H_2_O.

qPCR was performed using a Stratagene Mx3000P thermocycler (Agilent Technologies). Each 20 μL reaction included 10 μL of 2× Taq Master Mix, 2 μL of primer-probe mixture (reverse primer (RP):forward primer (FP):probe at 4:4:1, each at 1 μM in H_2_O), and 8 μL of diluted ligated product. The thermal profile involved an initial denaturation at 95°C for 3 min, followed by cycles of 95°C for 5 s and 60°C for 1 min.

For titration, SARS-CoV-2 S1 protein stock was diluted in DPBS with Ca^2+^ and Mg^2+^ to concentrations of 1000 nM, 100 nM, 10 nM, 1 nM, 0.1 nM, 0.01 nM, and 0.001 nM, using low-bind tubes and tips, as described previously. Each 2 μL dilution was added to the folded aptamer pair mixture (AptS1-tSP4 with 5′-PO4/XN-268s with 3′-OH), and the incubation, ligation, and qPCR steps were performed as described for aptamer pair screening.

### Identification of an epitope peptide binding to AptS1-tSP10 aptamer using nLC-MS/MS

Sample preparation, proteomic data acquisition, and data analysis were carried out as described previously.^16^ The proteomic analysis was performed using an UltiMate 3000 nanoRSLC (Dionex, Thermo Fisher Scientific, Mississauga, ON, Canada) and an Orbitrap Fusion mass spectrometer (Thermo Fisher Scientific, Mississauga, ON, Canada).

### MD simulation

The secondary structure of the AptS1-tSP10 aptamer was predicted using the mFold web server. SimRNA and VMD programs were used to obtain a corresponding 3D structure of the aptamer. Molecular dynamic simulations of the aptamer, target S1 protein, and their complexes were performed with the GROMACS-2021 program package. The Amber14sb force field and the TIP3P water model were used. Simulations were carried out in cubic periodic boxes. All systems were solvated and neutralized, containing additional Na^+^ and Cl^−^ ions to mimic the 0.15 M salt concentration in the binding buffer. MD simulations of 200 ns were performed at constant particle number (N), pressure (P), and temperature (T) (NPT ensemble) at 300 K and 1 atm, using the velocity-rescaling thermostat and the Parrinello-Rahman barostat. Clustering analysis of the MD trajectories was performed using the Clustering plugin in VMD. The HADDOCK web server was used for aptamer/protein docking. The peptide sequence named SA10 was used as a binding site in the docking simulations to evaluate its interaction with the AptS1-tSP10 aptamer. Quantum chemical calculations were performed using the fragment molecular orbital (FMO) method, which speeds up and simplifies calculations of large biomolecules by dividing the system into smaller fragments. The target protein and the aptamer were represented as 661 and 40 fragments, respectively. Geometry optimization and the calculation of pair interaction energies (PIEs) were performed with third-order density functional tight-binding (DFTB3) using 3ob parameters, D3(BJ) empirical dispersion, and the conductor-like polarizable continuum model of solvation (C-PCM). All calculations were conducted with the GAMESS(US) program.

## Data and code availability

The authors confirm that the data supporting the findings of this study are available within the article and its supplementary material. Additional raw data that support the findings of this study can be made available upon reasonable request from the corresponding author.

## Acknowledgments

M.V.B. thanks the 10.13039/501100000024Canadian Institutes of Health Research grant OV1-170353 for providing financial support. Molecular modeling was supported by an FWES-2025-0029 for О.S.K.

## Author contributions

S.P. designed experiments, selected aptamers, performed binding affinity assays, and collected and analyzed the data. P.V.A., I.A.S., D.M., A.V.R., F.N.T., and A.S.K. designed the software, conducted molecular dynamics and quantum mechanics studies, and analyzed the data. E.Y., S.U., and J.P.P. produced pseudoviruses. E.Y. performed BLI assays with the pseudoviruses. A.J., G.K.U., Y.G., and B.P.L. performed affinity assays. A.R. and N.M. performed PLA-qPCR experiments, collected, and analyzed the data. Z.M., A.K., and N.H. processed peptide samples using nLC-MS/MS and collected the data. S.P., E.Y., S.U., P.V.A., and A.R. wrote the manuscript. S.P., P.V.A., A.S.K., A.R., and Z.M. contributed to reviewing and editing the manuscript. M.V.B. supervised the study and oversaw funding acquisition. All authors reviewed the manuscript.

## Declaration of interests

M.V.B. serves as a Section Editor of *Molecular Therapy-Nucleic Acids.*

## Supplemental information

Document S1. Figures S1–S5 and Tables S1–S4

Document S2. Article plus supplemental information
