# Supplementary material for: Aptamer-based approaches for sensitive detection and epitope mapping of SARS-CoV-2 spike protein
Source: Mol Ther Nucleic Acids. 2025 Dec 9;37(1):102790. doi: 10.1016/j.omtn.2025.102790 (PMC12800406; doi:10.1016/j.omtn.2025.102790)

OMTN, Volume *37*

Supplemental information

Aptamer-based approaches

for sensitive detection and epitope mapping

of SARS-CoV-2 spike protein

Suttinee Poolsup, Elnaz Yaghoobi, Aliaksandra Radchanka, Nandanee Mulloo, Spencer Uguccioni, John Paul Pezacki, Abdullah Khraibah, Aasha Jawad, Gurcharan K. Uppal, Yuxuan Gu, Benjamin Patrick Lapointe, Nico Hüttmann, Zoran Minic, Polina V. Artyushenko, Irina A. Shchugoreva, Anastasia V. Rogova, Felix N. Tomilin, Dmitry Morozov, Anna S. Kichkailo, Olga S. Kolovskaya, and Maxim V. Berezovski

Supplemental Information

Table S1. Full-length sequences (80 nt) of the selected aptamer binding to the SARS-CoV-2 S1 protein. The enriched sequences were obtained through NGS data enrichment. The primer-binding regions are highlighted in bold.

**Aptamer**	**Aptamer sequence**
S1-SP1	**CTCCTCTGACTGTAACCACG**AACCACGTATTGTGCACTGTCTCATTAGTAATCGCTCCCA**GCATAGGTAGTCCAGAAGCC**
S1-SP2	**CTCCTCTGACTGTAACCACG**GCGCAAGCCGGGGTGTATGTGTTATACGTGCGTGTATCGA**GCATAGGTAGTCCAGAAGCC**
S1-SP3	**CTCCTCTGACTGTAACCACG**GCGCAAGCCGGGGTGTACGCGTTATACGTGCGTGTATCGA**GCATAGGTAGTCCAGAAGCC**
S1-SP4	**CTCCTCTGACTGTAACCACG**TAATGCCTAACTCTTTTTGTGTTTGCGATCTTTGCACATA**GCATAGGTAGTCCAGAAGCC**
S1-SP5	**CTCCTCTGACTGTAACCACG**TATCTCCGCACCGGCATGGAACGACAGATTCCCAACTTCC**GCATAGGTAGTCCAGAAGCC**
S1-SP6	**CTCCTCTGACTGTAACCACG**CGGTTACGATCGGGTTACAAACACGGCTGACACGAATTCA**GCATAGGTAGTCCAGAAGCC**
S1-SP7	**CTCCTCTGACTGTAACCACG**TCCTCATCCCGCAAACCGCCTTGTCAGAAGAACTTTGCAT**GCATAGGTAGTCCAGAAGCC**
S1-SP8	**CTCCTCTGACTGTAACCACG**TTCCATTTTACGGATCCCCTGGTGTTATAGAATGGTGTAT**GCATAGGTAGTCCAGAAGCC**
S1-SP9	**CTCCTCTGACTGTAACCACG**TTTCTTTCGCGTGTGGTCTCAAGAAGAGTACCGTGACTGC**GCATAGGTAGTCCAGAAGCC**
S1-SP10	**CTCCTCTGACTGTAACCACG**CACACTTTCTGCCCGCCTTCTCCCTCCGTTCCCCTCCCCG**GCATAGGTAGTCCAGAAGCC**
S1-SP11	**CTCCTCTGACTGTAACCACG**ATGTCCTCGCACACCCAAACGCACTCATCTCCCCACCCAT**GCATAGGTAGTCCAGAAGCC**
S1-SP12	**CTCCTCTGACTGTAACCACG**ATTCATGCGCCAATAGTGGTTTGGAAATGTCTCCCCATAC**GCATAGGTAGTCCAGAAGCC**
S1-SP13	**CTCCTCTGACTGTAACCACG**TATCGCGTCATTCGATCCATTTGTACATCATTGTGCATAG**GCATAGGTAGTCCAGAAGCC**
S1-SP14	**CTCCTCTGACTGTAACCACG**TCTTTACAAGTTCACACCCTTGGTACATGACTACATTCAC**GCATAGGTAGTCCAGAAGCC**

Table S2. Hydrogen bonds in the S1p/AptS1-tSP10 complexes. The amino acids in the SA10 binding site are highlighted in bold.

S1p-cl0/AptS1-tSP10		S1p-cl1/AptS1-tSP10	
Nucleotide	Amino acid	Nucleotide	Amino acid
DC-24	ARG-346	DC-26	ARG-346
DC-26	SER-349	DG-28	TYR-351
DC-16	LYS-444	DC-39	ARG-403
DT-21	LYS-444	DC-24	LYS-444
DC-22	LYS-444	**DC-16**	**GLY-447**
**DT-21**	**GLY-446**	DG-28	THR-470
**DT-25**	**ASN-448**	DC-33	GLN-493
**DG-15**	**ASN-450**	DG-40	SER-494
**DT-25**	**ASN-450**	DC-39	TYR-505
DC-27	ARG-466		
DG-40	GLN-493		
DG-40	SER-494		

Table S3. Total interaction energy (kcal mol $^{-1}$) and pair interaction energies (PIEs, kcal mol $^{-1}$) for S1p/AptS1-tSP10 complexes. List of strong contacts between the aptamer and the S1 protein in both conformations. The blue color indicates dispersion as the dominant attractive interaction; the pink color indicates electrostatic interaction. The amino acids in the SA10 binding site are highlighted in bold.

S1p-cl0/AptS1-tSP10			S1p-cl1/AptS1-tSP10		
Total interaction energy, kcal/mol: -277.13			Total interaction energy, kcal/mol: -254.73		
Nucleotide	Amino acid	PIEs, kcal mol $^{-1}$	Nucleotide	Amino acid	PIEs, kcal mol $^{-1}$
DC22	LYS444	-26.81	DC23	LYS444	-27.09
DC16	LYS444	-26.78	DC27	THR470	-24.756
DC26	ARG466	-25.77	DC26	ARG346	-21.478
DC23	ARG346	-22.79	DC27	TYR351	-20.054
DT21	LYS444	-19.10	DC38	ARG403	-17.183
DC23	LYS444	-17.98	DC38	TYR505	-16.038
DC26	SER349	-16.00	DC37	ASN165	-13.512
**DT21**	**GLY446**	**-12.13**	**DC16**	**GLY447**	**-9.606**
DC26	ALA352	-7.38	DC33	GLN493	-7.992
DG40	SER494	-7.17	**DG15**	**TYR449**	**-7.605**
DC24	ARG346	-6.90	DG40	TYR495	-6.775
DC39	GLN493	-6.70	**DC27**	**ARG454**	**-6.752**
**DT25**	**ASN450**	**-6.61**	DC27	LEU492	-5.721
**DG40**	**TYR449**	**-6.58**	DG40	TYR505	-5.171
**DC26**	**TYR451**	**-5.12**	**DC26**	**ASN450**	**-4.477**
DT21	VAL445	**-5.07**	DC39	ARG403	-4.43
DC26	ALA348	-4.96	DC38	GLY232	-4.063
DG40	GLN493	-4.90	DG40	GLY496	-3.556
**DG15**	**ASN450**	**-4.75**	DG40	ARG403	-3.33
**DC16**	**GLY446**	**-4.71**	**DC16**	**TYR449**	**-3.05**
**DC20**	**VAL445**	**-4.08**			
**DT25**	**ASN448**	**-4.08**			

Table S4. List of aptamer sequences used for proximity ligation. It includes aptamers (AptS1-tSP4, AptS1-SP10, AptS1-SP11 – selected in this study, and XN-268s – selected by Shi et al.) modified for proximity ligation. Aptamer sequences (black, bold) are modified with a linker complementary fragment (green). All reactions used the same 20-nt linker and Cy5-labeled probe. Six aptamer pairs were tested: three pairs with XN-268s with 5′-PO_4 and one of the remaining aptamers with 3′-OH, and three pairs with XN-268s with 3′-OH and one of the remaining aptamers with 5′-PO_4.

**Aptamer**	**Variant**	**Sequence (5'→3')**	**Primer (5'→3')**
AptS1-tSP4	5′-PO_4	CTCCTCTGACTGTAACCACG**TAATGCCTAACTCTTTTTGTGTTTGCGATCTTTGCACATA**	RP: CGCAAACACAAAAAGAGTTAGGCA
	3′-OH	**TAATGCCTAACTCTTTTTGTGTTTGCGATCTTTGCACATA**GCATAGGTAGTCCAGAAGCC	FP: TGCGATCTTTGCACATAGCA
AptS1-tSP10	5′-PO_4	CTCCTCTGACTGTAACCACG**CACACTTTCTGCCCGCCTTCTCCCTCCGTTCCCCTCCCCG**	RP: CGGGCAGAAAGTGTGCGT
	3′-OH	**CACACTTTCTGCCCGCCTTCTCCCTCCGTTCCCCTCCCCG**GCATAGGTAGTCCAGAAGCC	FP: TTCTCCCTCCGTTCCCCT
AptS1-tSP11	5′-PO_4	CTCCTCTGACTGTAACCACG**ATGTCCTCGCACACCCAAACGCACTCATCTCCCCACCCAT**	RP: TTGGGTGTGCGAGGACAT
	3′-OH	**ATGTCCTCGCACACCCAAACGCACTCATCTCCCCACCCATGCA**TAGGTAGTCCAGAAGCC	FP: CTCATCTCCCCACCCATGCA
XN-268s	5′-PO_4	CTCCTCTGACTGTAACCACG**CACGCATAACCGAGCTGGGGTGGGGTAGTGGTATGGAGCGTCAGTTGTTATGCGTG**	RP: AACTGACGCTCCATACCACTAC
	3′-OH	**CAGCACCGACCTTGTGCTTTGGGAGTGCTGGTCCAAGGGCGTTAATGGACA**GCATAGGTAGTCCAGAAGCC	FP: GGTAGTGGTATGGAGCGTCA
Linker		TCAGAGGAGGGCTTCTGG	
Probe		/5Cy5/AGTCCAGAA/TAO/GCCCTCCTCTGACTG/3IAbRQSp/	

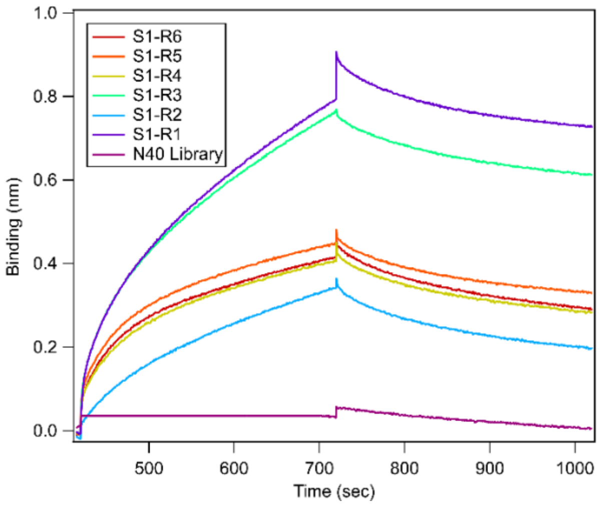

Figure S1. Affinity screening test of enriched DNA pools after SELEX. The binding evaluation of six enriched DNA pools was obtained through asymmetric-ePCR SELEX targeting SARS-CoV-2 S1 subunit proteins, compared to an initial DNA N40 library using BLI. The BLI sensorgrams display the association and dissociation curves of the six enriched pools derived from the aptamer selection targeting the S1 protein of SARS-CoV-2.

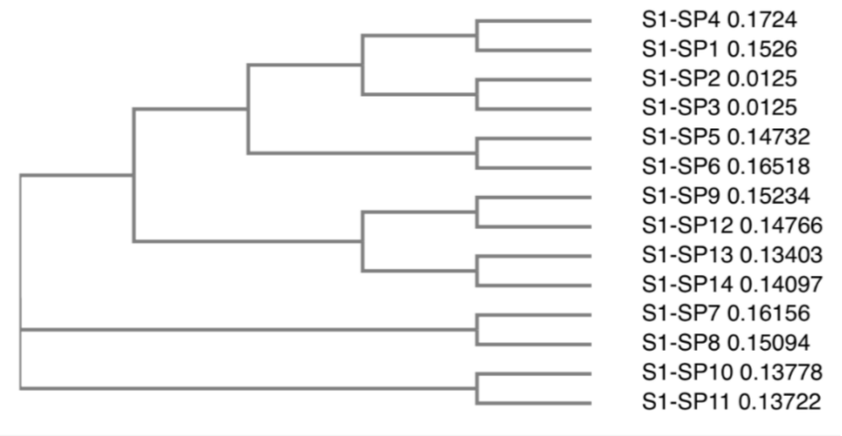

Figure S2. Phylogenetic tree of S1-binding DNA aptamers. The diagram created from Clustal Omega shows the three main groups of fourteen enriched aptamer sequences targeting the SARS-CoV-2 S1 subunit of the spike protein, with phylogenetic distances indicating the number of pairwise nucleotide differences between closely related sequences. The sequences were obtained from the count-enrich FASTAptamer.

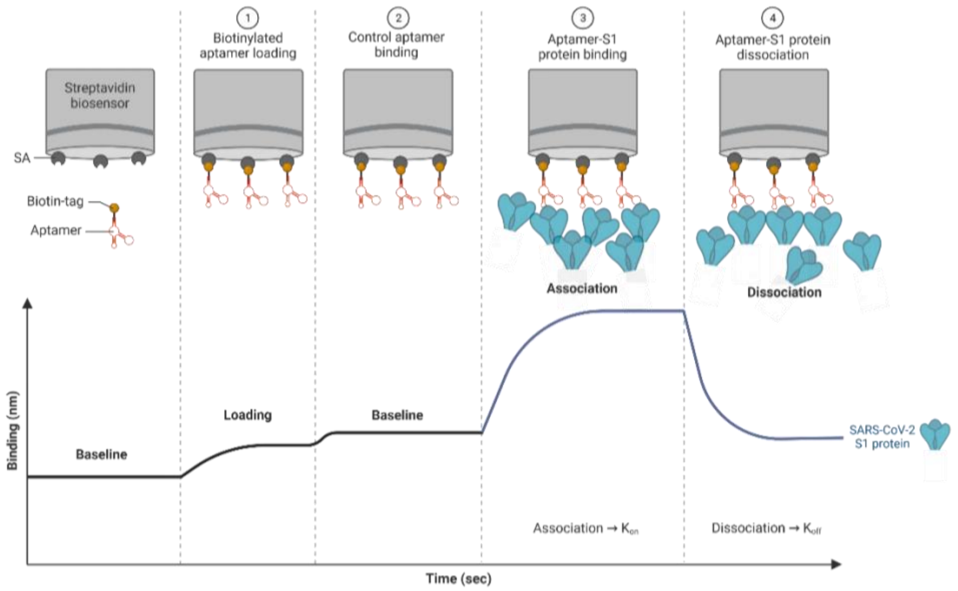

Figure S3. Schematic BLI workflow for aptamer-protein affinity tests. The affinity screening of enriched aptamers targeting the S1 protein involved labeling each aptamer with biotinylated forward or reverse primers at different times. These aptamers were then immobilized on the streptavidin biosensor before loading the S1 protein in the association step on the aptamer-coated streptavidin biosensor.

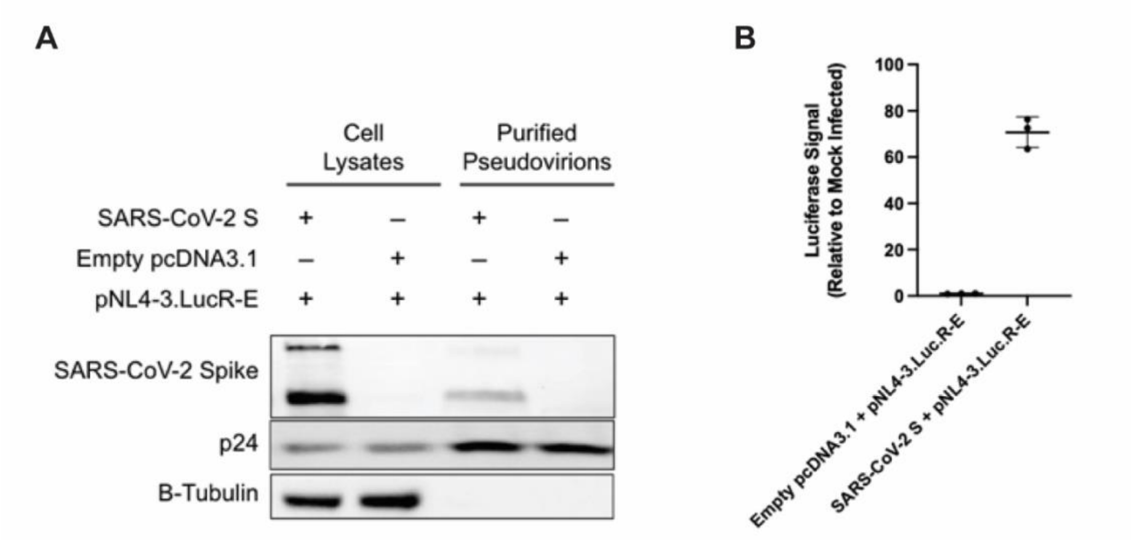

Figure S4. Production and infectiousness of SARS-CoV-2 pseudovirions. (A) Western blot analysis of lysates from HEK293T cells transfected with plasmids encoding the SARS-CoV-2 S and pNL-4-3.LucR-E, compared to negative control cells transfected with empty pcDNA3.1 and pNL4-3.LucR-E. Pseudovirions in the supernatants of the transfected HEK293T cells were purified by ultracentrifugation in a 30% sucrose cushion. (B) Luciferase assay of Huh7 cells transduced with 100μL of supernatant from HEK293T cells transfected with the indicated plasmids. Dots represent biological replicates of n=3 experiments, and error bars show the standard error.

Figure S5. Screening of aptamer pairs selected in this study (AptS1-tSP4, AptS1-tSP10, and AptS1-tSP11). The tested aptamer pairs did not show a significant difference in Ct values in the presence or absence of the S1 protein. This result may indicate that the aptamers either compete for the same binding site or are positioned too far apart to allow efficient ligation.

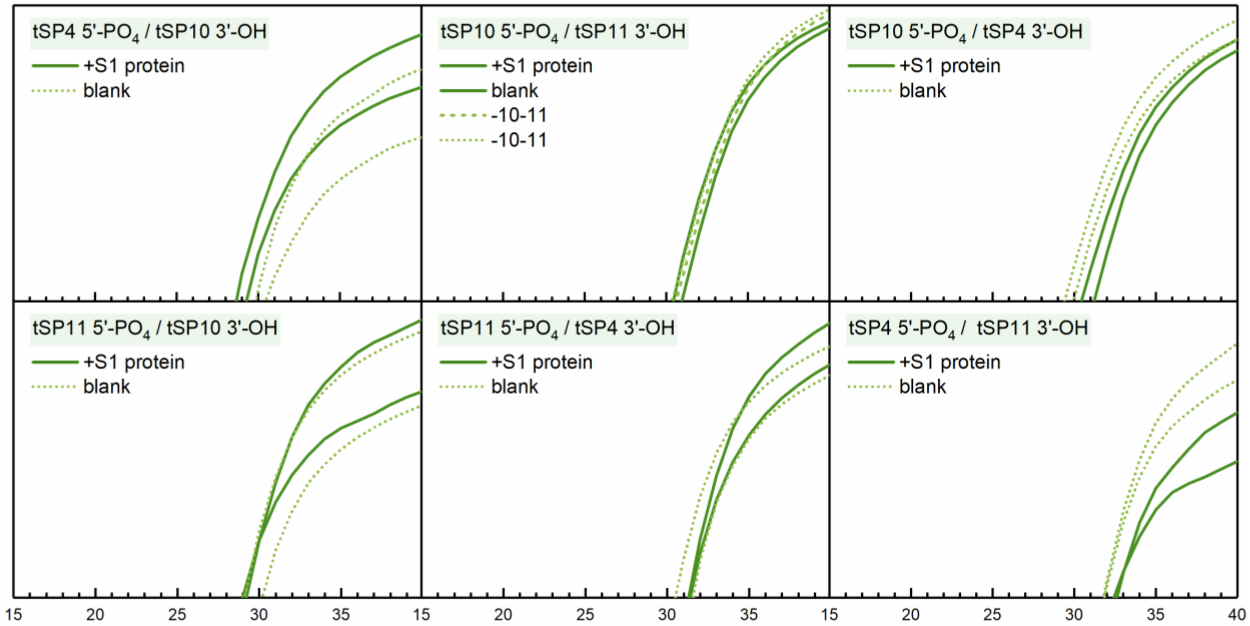

Supplement: Document S1. Figures S1–S5 and Tables S1–S4 [file mmc1.pdf]
